# Cure of Glanders in Man

**Published:** 1843-05

**Authors:** 


*Cure of Glanders in Man.*—A WAGONER, nineteen years of age, entered the Hopital de la Charite, in Paris, on the 18th of October, 1831. He complained of having felt ill for the week preceding, without being able to specify any particular seat of disease. Soon intense pains were felt in the ankle and knee-joints, and the muscles of the leg and thigh, though unattended with swelling or redness. His pulse became quick, thirst intense; headach and prostration. On the 25th of October pustules, filled with a purulent matter appeared on the instep and upper surface of the three smaller toes of the left foot. These pustules broke, and cicatrisation was completed in a few days; but a diffused swelling now made its appearance in the anterior part of the superior third of the thigh, followed by two similar tumours, one on each leg. Mouneret, under whose care the patient was placed, now suspected the nature of the disease, and ascertained that one of the horses kept in the stable where the patient had been sleeping actually had the glanders. For the eight months ensuing tumours of a similar kind to the foregoing were successively and incessantly appearing on all parts of the upper and lower extremities, though they continued one after another to disperse, and nothing in the general condition of the patient, except his emaciation, gave cause for alarm; yet one curious collateral circumstance is stated. Early in December, 1841, a horse being inoculated with the matter from one of the abscesses, died in the course of five days, without, however, presenting during life any of the ordinary symptoms, or after death any of the usual morbid appearances belonging to the disease.

The treatment of the patient was the same nearly throughout, consisting chiefly of decoction and extract of cinchona in large doses, with wine.

On the 5th of July, 1842, iodine with iodide of potassium was administered. This was followed by an attack of erysipelas in the left arm, and the iodine was suspended, to be resumed on the 17th. No new tumours had appeared during the previous two months; the cicatrisation of those still existing was soon afterwards completed, and the patient was discharged perfectly cured on the 31st of July.

Andral, and other able pathologists who saw this case, were unanimous in pronouncing it a true instance of glanders. The journal from which we have extracted the above relation says—"The case is unique. In all the instances of glanders in the human subject reported hitherto the disease has proved fatal."—*Gazette des Hôpitaux.*—*Lond. Lancet.*

